# Toripalimab induces exacerbation of psoriasis in an esophageal cancer patient: a case report

**DOI:** 10.3389/fonc.2025.1596818

**Published:** 2025-08-27

**Authors:** Luping Zhao, Zhen Cheng, Piaopiao Li

**Affiliations:** ^1^ Department of Pharmacy, Dongyang People’s Hospital, Dongyang, Zhejiang, China; ^2^ Department of Medical Oncology, Dongyang People’s Hospital, Dongyang, Zhejiang, China

**Keywords:** toripalimab, esophageal cancer, psoriasis, case report, adverse effects

## Abstract

**Background:**

Toripalimab in combination with cisplatin and paclitaxel is indicated as a first-line treatment for metastatic esophageal squamous cell carcinoma (ESCC). Toripalimab, a PD-1 inhibitor, commonly causes immune-related adverse reactions such as immune-related rash, immune-related pneumonitis, and immune-related diarrhea and colitis. This study reports a case of toripalimab-induced exacerbation of psoriasis in an esophageal cancer patient, emphasizing the need for careful monitoring and management in such treatments.

**Case presentation:**

A 60-year-old male patient with a history of well-controlled psoriasis without medication and no other significant medical conditions was hospitalized due to recurrence and exacerbation of psoriasis following two cycles of Toripalimab combined with cisplatin and nab-paclitaxel therapy after esophageal malignancy surgery. The psoriasis flare-up was considered to be induced by Toripalimab. After admission, the patient was treated with high-dose methylprednisolone. Upon symptom control, the steroid dosage was gradually tapered and completely discontinued over a total course of approximately eight weeks, with no recurrence throughout the tapering and discontinuation period.

**Conclusion:**

For cancer patients with pre-existing autoimmune diseases, particularly those with psoriasis, comprehensive multidisciplinary evaluation is essential before initiating Toripalimab therapy. During immunotherapy cycles, specialized follow-up by dermatologists and clinical pharmacists is recommended to monitor the status of pre-existing dermatological conditions and potential adverse reactions.

## Introduction

Toripalimab, the first domestically developed PD-1 inhibitor in China, has become a crucial therapeutic option for advanced malignancies including melanoma and esophageal squamous cell carcinoma since its approval in China in 2018 (1). Toripalimab is a humanized IgG4 monoclonal antibody that specifically binds to the PD-1 receptor on the surface of T cells. By blocking the interaction between PD-1 and its ligands PD-L1/PD-L2 on tumor cells, it alleviates the immunosuppressive effects of the tumor microenvironment, thereby restoring T cells’ ability to recognize and eliminate tumor cells (2, 3).

Psoriasis is a chronic, immune-mediated inflammatory skin disease (4). A systematic, worldwide review found the prevalence of psoriasis ranged from 0.5 to 11.4 percent in adults and 0 to 1.4 percent in children (5). The etiology of psoriasis is multifactorial, with medication exposure being a significant external contributing factor (6). Currently, case reports have indicated that nivolumab, pembrolizumab, durvalumab, and atezolizumab may induce or exacerbate psoriasis (7).While the specific mechanisms underlying ICI-induced psoriasis remain controversial, the overexpression of Th1/Th17-specific cytokines, such as IL-17, following blockade of PD-1 may be involved (8).Our case provides the first documented evidence of Toripalimab-induced psoriasis flare in a patient with esophageal cancer. This case underscores the need to screen for psoriasis history before Toripalimab initiation, as pre-existing autoimmune conditions may heighten flare risk. Clinicians should monitor psoriasis patients on toripalimab for paradoxical worsening, even if baseline disease is quiescent.

## Case description

We describe a 60-year-old male patient presenting with progressive postprandial dysphagia. Gastroscopic examination confirmed esophageal malignancy, while contrast-enhanced chest CT demonstrated: (1) carcinoma at the lower esophagus with periesophageal and mediastinal lymphadenopathy; (2) multiple subcentimeter lymph nodes in bilateral supraclavicular regions, lesser omental sac, and retroperitoneum, raising suspicion of metastatic dissemination. On October 16, 2024, the patient underwent “radical esophagectomy via a right thoracic approach combined with laparotomy” under general anesthesia. The postoperative pathological findings of the patient indicate: (Esophagus, lower segment) Medullary type squamous cell carcinoma (tumor size: 2.4×2×2 cm) with moderate differentiation. The tumor infiltrates into the superficial muscular layer, with visible vascular tumor emboli and perineural invasion. Lymph node metastasis: 10/24 lymph nodes showed metastatic carcinoma: perigastric: 4/11 nodes, para-azygous: 0/1 node, diaphragmatic crus: 1/4 nodes, inferior pulmonary vein: 1/1 node, subcarinal: 4/7 nodes. Both proximal and distal resection margins are negative. Final pathological staging: pT2N3Mx. The patient had a 40-year history of alcohol consumption and a 5-year smoking history, and had abstained from both smoking and alcohol since undergoing surgery. The patient had a 20-year history of psoriasis, yet no psoriatic plaques were documented during the baseline dermatological assessment prior to ESCC treatment initiation. The patient had no other significant past medical history, with hepatic and renal functions otherwise within normal limits.

**Figure 1:**
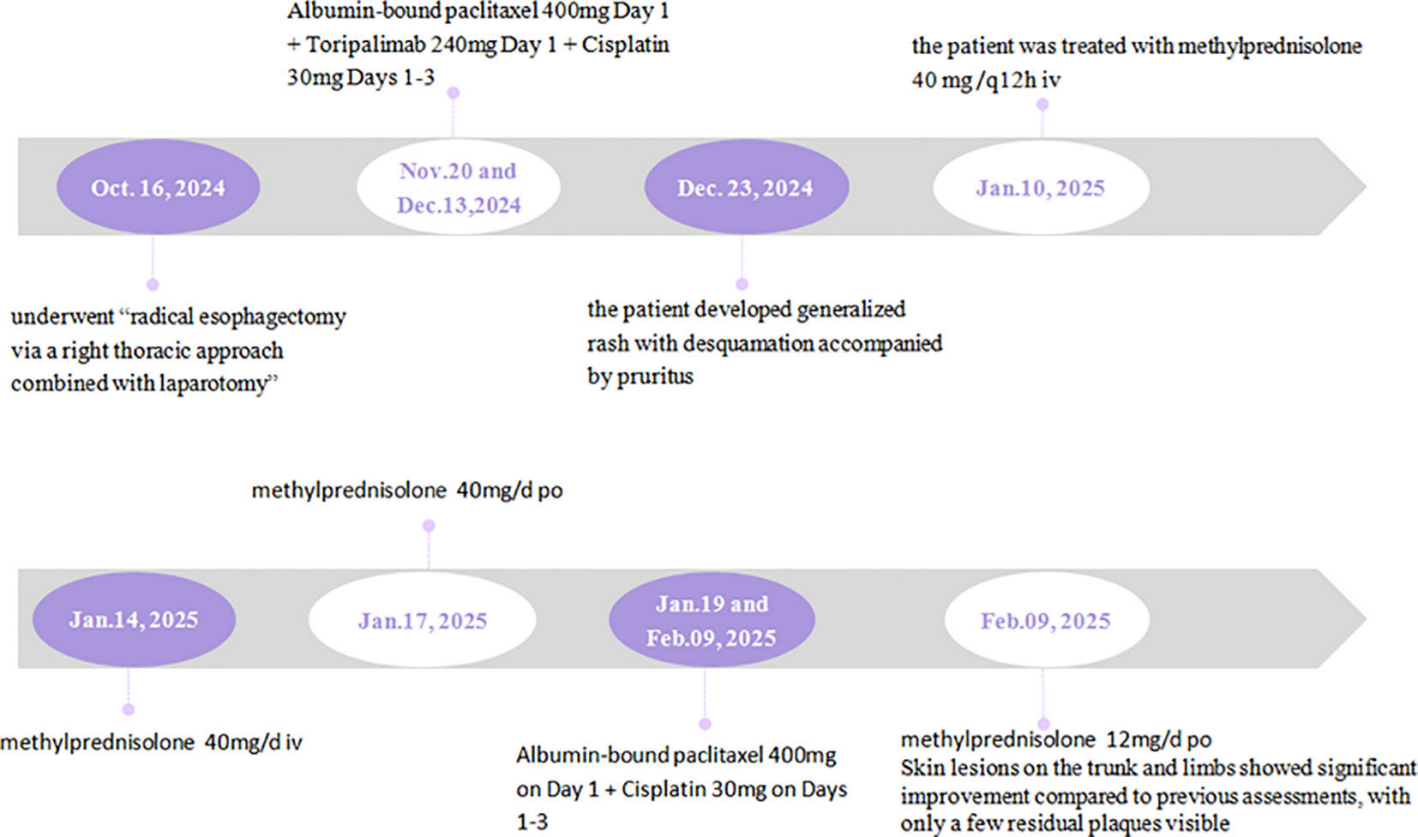
Timeline.

Baseline evaluation prior to treatment initiation revealed a body mass index of 20.82 kg/m², Nutritional Risk Screening (NRS) 2002 score of 2 (indicating moderate nutritional risk), and Eastern Cooperative Oncology Group (ECOG) performance status grade 1 (fully ambulatory).The patient initiated combination therapy with Albumin-bound paclitaxel 400mg Day 1 + Toripalimab 240mg Day 1 + Cisplatin 30mg Days 1–3 on November 20, 2024, and December 13, 2024. During chemotherapy, the patient received comprehensive supportive care including omeprazole for stress ulcer prophylaxis, compound glycyrrhizin for hepatoprotection, ondansetron and/or metoclopramide as antiemetic agents, and human granulocyte colony-stimulating factor (G-CSF) for neutropenia prophylaxis. Approximately 10 days after the completion of the second cycle, the patient developed generalized rash with desquamation accompanied by pruritus, but no pain. The dermatologist prescribed Xiaoyin Capsules, Compound Glycyrrhizin, and Halometasone and Triclosan Cream to alleviate symptoms. However, the symptoms showed inadequate improvement, and the patient was subsequently hospitalized for further treatment on January 10, 2025. **Figure 1** provides a comprehensive illustration of the patient’s detailed treatment process.

**Figure 2:**
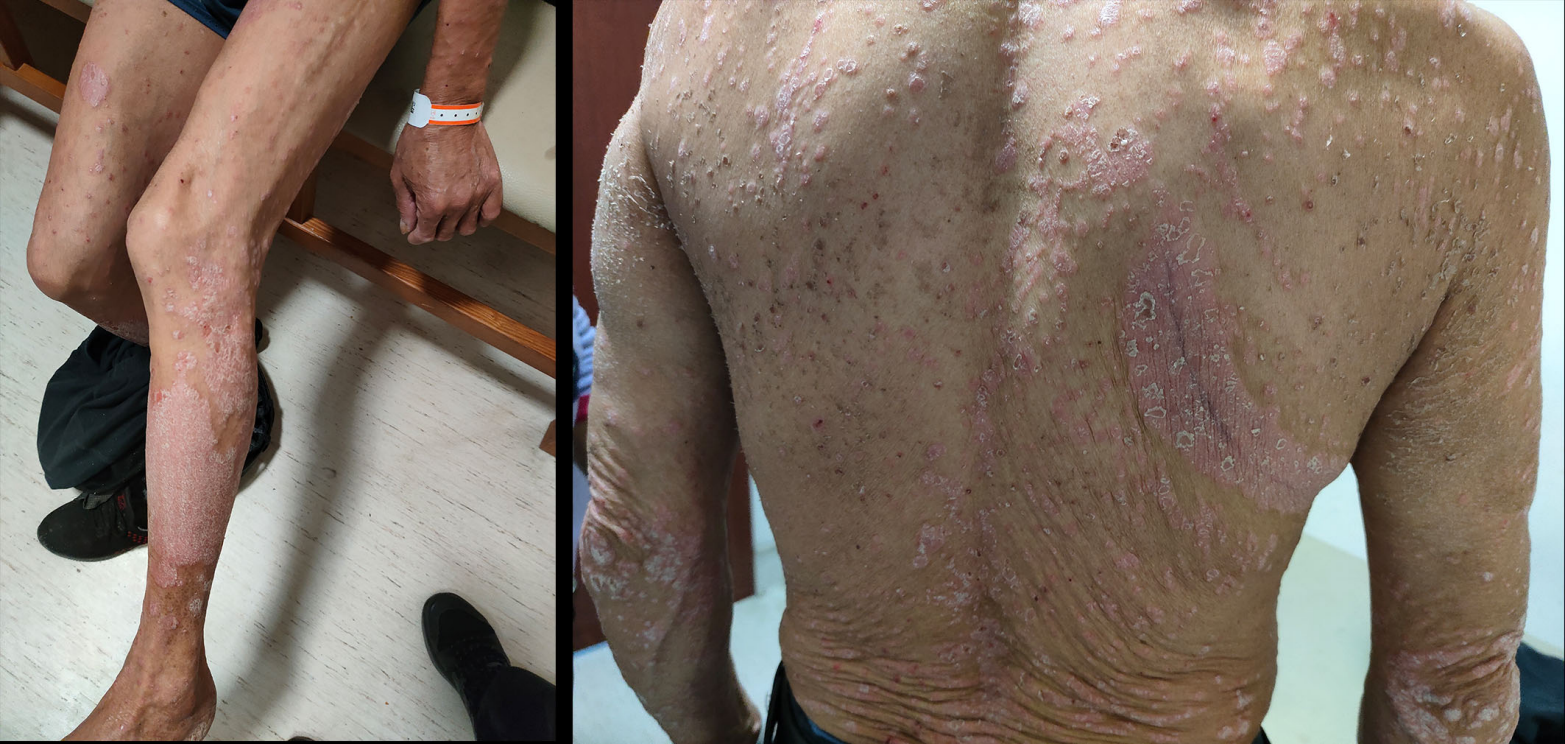
The patient’s symptoms upon hospital admission.

Upon hospital admission, the patient was conscious and alert, with vital signs including temperature and blood pressure within normal ranges. Multiple plaques of varying sizes, well-demarcated, irregularly shaped, and erythematous were observed on the trunk and extremities, covered with thick scales. Some lesions coalesced into patches, with a positive Auspitz sign. The patient reported pruritus but no pain (**Figure 2**). Based on the clear clinical presentation, the patient was diagnosed with an exacerbation of psoriasis vulgaris. According to the patient’s medication history and the Naranjo Adverse Drug Reaction Probability Scale, it has been concluded that the exacerbation is attributed to toripalimab. The patient was treated with methylprednisolone 40 mg every 12 hours for 4 days, in accordance with the guidelines for immune checkpoint inhibitor-related adverse events (9, 10). The systemic desquamative rash and pruritus showed improvement compared to previous status. The treatment was then switched to methylprednisolone 40 mg once daily for 3 days, followed by gradual tapering to a maintenance dose of methylprednisolone 20 mg once daily orally.

**Figure 3:**
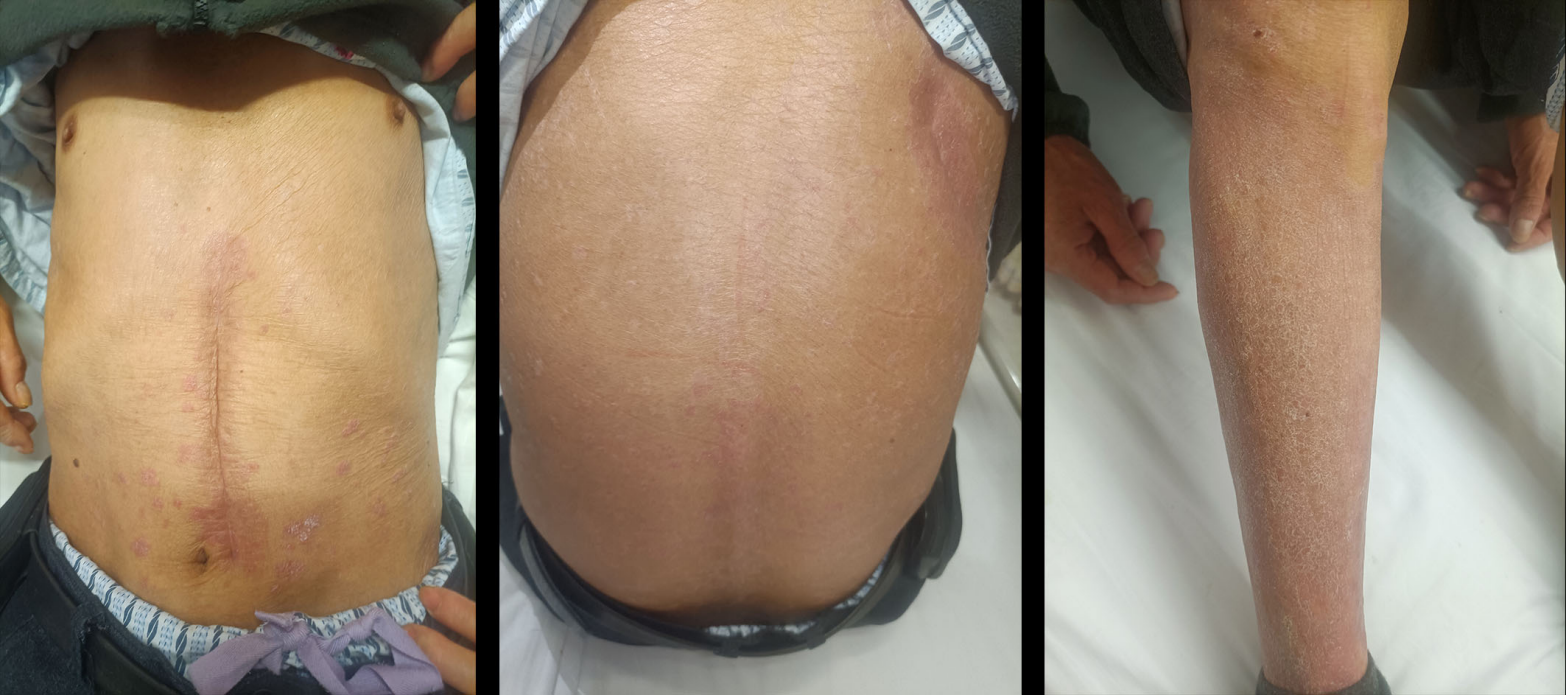
The patient exhibited notable alleviation of symptoms following a month-long treatment.

After exclusion of chemotherapy contraindications, the patient received chemotherapy regimens on January 19, 2025 and February 9, 2025 as follows: Albumin-bound paclitaxel 400mg on Day 1 + Cisplatin 30mg on Days 1-3. The immunotherapy was ceased in view of the psoriatic flare. The patient reported no discomfort during treatment. Upon admission on February 9, the dose of methylprednisolone had been tapered to 12mg once daily. Skin lesions on the trunk and limbs showed significant improvement compared to previous assessments, with only a few residual plaques visible (as shown in **Figure 3**). The patient successfully discontinued methylprednisolone through a stepwise tapering protocol without psoriasis recurrence during the withdrawal period or subsequent surveillance.

## Discussion

Per Naranjo criteria, the likelihood of drug-induced psoriasis exacerbation was graded as probable for toripalimab (score=5) and possible for both albumin-bound paclitaxel and cisplatin (scores=1 each). We hypothesize that toripalimab was the trigger to the psoriasis exacerbation in our patient.

Immune checkpoint inhibitors (ICIs) are a groundbreaking advancement in cancer treatment, offering patients hope through their remarkable effectiveness. However, it is essential to recognize that, alongside these significant benefits, ICIs can lead to a range of immune-related side effects (irAEs). Non-specific maculopapular rash, pruritus, lichenoid reactions, eczema, and vitiligo are the most common dermatologic side effects (11). It has been reported that ICI-associated psoriasis or psoriasiform reactions accounted for 3.8% of all cutaneous toxicities in the EudraVigilance drug safety database (12). There have been increasing reports of cases where ICIs nivolumab and pembrolizumab triggered or exacerbated psoriasis (13–18). In the review by Liliana Gabriela Popa, in addition to nivolumab and pembrolizumab, durvalumab and atezolizumab have also been reported to induce or exacerbate psoriasis (19).

In a systematic review of 242 cases of ICI-mediated psoriasis (including five cases with the inverse phenotype), the mean numbers of ICI cycles prior to ICI-induced *de novo* or flare of pre-existing psoriasis were 9.9 cycles and 6.4 cycles, respectively (20). However, this case report shows that the patient developed severe psoriasis exacerbation after completing two cycles of toripalimab therapy. We hypothesized a potential correlation between the patient’s chronic alcohol consumption and smoking history. Accumulating clinical evidence has established smoking as a significant independent risk factor for psoriasis recurrence (P<0.05) (21). Furthermore, longitudinal cohort studies demonstrated that various smoking phenotypes - particularly smoking initiation age, cigarettes-per-day intensity, and cumulative pack-years-consistently show dose-dependent positive associations with psoriasis risk (OR>1,P<0.05) (22). Clinical evidence confirms a dose-dependent positive association between alcohol consumption and psoriasis incidence, with high-dose intake (>45 g/day) significantly elevating disease susceptibility (23).Given the patient’s brief abstinence duration (only 1 month smoking/alcohol cessation) prior to toripalimab initiation, it is mechanistically plausible that pre-existing alcohol and tobacco exposure histories may have potentially exacerbated psoriatic progression during anti-PD-1 therapy. Analysis of concomitant medications during the patient’s chemotherapy revealed that G-CSF may contribute to immune evasion by suppressing T-cell function and promoting differentiation of immunosuppressive cells (24, 25). Given that psoriasis is a Th17-mediated autoimmune disorder, this mechanism theoretically carries the potential to exacerbate psoriatic inflammation. However, there is a lack of direct clinical evidence.

Although a possible rationale for the pathogenesis can be proposed, this remains speculative. Psoriasis is recognized as a T-cell- and dendritic-cell-mediated disease. IL-17 and IL-22 produced by T helper (Th) 1, 17, and 22 cells play a central role in its pathophysiology (26).The PD-1 pathway physiologically downregulates Th cell activity. PD-1 inhibitors exert their effects by blocking PD-1/PD-L1 interactions, thereby reversing T cell suppression and resulting in hyperactivation of psoriasis-associated Th1/Th17 subsets accompanied by dysregulated cytokine production (27).These activated T cells secrete various cytokines including interferon-γ, IL-1, IL-17, and IL-22 (28),which subsequently migrate to cutaneous tissues and activate resident immune cells (keratinocytes, dendritic cells, and neutrophils), ultimately inducing or aggravating psoriatic lesions (29).

Upon IL-17 stimulation, keratinocytes exhibit effector functions by autonomously releasing VEGF, S100 proteins, antimicrobial peptides, and chemokines. This cytokine milieu facilitates the recruitment of additional immune cells (e.g., neutrophils) and establishes a self-perpetuating inflammatory cascade that sustains psoriatic plaques (30, 31). Pharmacological inhibition of the PD-1 pathway specifically enhances Th17 responses (32), providing a mechanistic explanation for psoriasis exacerbation observed during toripalimab therapy. Furthermore, PD-1 blockade-induced psoriasiform dermatitis is histologically characterized by prominent epidermal infiltration of CD8+ T cells and overexpression of IL-6, IL-23, and IL-17A cytokines (33). Martina Morelli also found that IL-6 and TNF-α are aberrantly expressed in psoriasis reactions induced by anti-PD-1, at levels comparable with chronic psoriasis (34). At the molecular level, TRIM14 upregulation promotes TRAF3 degradation via NDP52-mediated autophagy, relieving NF-κB pathway inhibition and exacerbating inflammation (35).The recurrence of psoriasis may be closely associated with tissue-resident memory T (Trm) cells persisting within clinically healed lesions. These cells are reactivated following immune checkpoint inhibitor (ICI) administration, secreting pro-inflammatory cytokines such as IL-17A/F, ultimately leading to recurrence in the original lesional areas (36, 37).Research shows that toripalimab binds to the FG loop of PD-1 with 12-fold higher affinity than pembrolizumab, resulting in enhanced T cell activation (including amplified Th1 and myeloid inflammatory responses) (38). This mechanism may potentially influence the intensity of irAEs.

The management of irAEs, as per the European Society for Medical Oncology (ESMO), Chinese Society of Clinical Oncology(CSCO) guidelines, is based on Common Terminology Criteria for Adverse Events (CTCAE) toxicity grading. For grades 3 toxicities, immunotherapy should be withheld until resolution to grade 0–1, with permanent discontinuation considered if no improvement occurs within 12 weeks. High-dose corticosteroids must be initiated promptly, and additional immunosuppressive agents (e.g., infliximab, mycophenolate mofetil, or cyclophosphamide) should be introduced if clinical deterioration persists under steroid therapy (39–41).

In our case, multiple erythematous plaques with varying sizes, well-demarcated borders, and irregular configurations were observed on the patient’s trunk and extremities, exhibiting thick, adherent scales. The Psoriasis Area and Severity Index(PASI)score was 21.8, with >30% body surface area (BSA) involvement. Grade 3 toxicity was confirmed by multidisciplinary evaluation, leading to toripalimab discontinuation and immediate intravenous methylprednisolone (40 mg every 12 hours). Post-treatment, the PASI score declined to 1.8 with <5% BSA involvement, demonstrating marked improvement.

Notably, the American Academy of Dermatology-National Psoriasis Foundation (AAD-NPF) opposes corticosteroid use for *de novo* or PD-1 inhibitor-exacerbated psoriasis due to risks of relapse during tapering and limited sustained efficacy (42).Kim A. Papp noted that glucocorticoids are commonly used in oncology to control early stages of irAEs, but not typically used by dermatologists to treat psoriasis because of long-term risks and the potential for disease flares upon glucocorticoids cessation (7).Given the strong endorsement of glucocorticoids for immune-related cutaneous toxicities by ESMO/CSCO/CCO/SITC guidelines, this intervention was prioritized. The patient achieved sustained remission without psoriasis recurrence after steroid withdrawal, supporting its selective utility in irAE management.

## Conclusion

To the best of our knowledge, this is the first evidence of psoriasis exacerbation in ESCC patients receiving toripalimab treatment. We suggest that prior to initiating PD-1/PD-L1, assessment for preexisting autoimmune conditions or family history of autoimmune disorders must be prioritized. Particularly for patients with documented psoriasis history, dermatologists should perform archival skin biopsies, while clinical pharmacists comprehensively evaluate prior immunomodulatory drug usage to verify completion of appropriate washout periods. During treatment, regular multidisciplinary follow-up is recommended to monitor cutaneous and systemic adverse reactions, with adverse event management strictly following CTCAE grading criteria. We will further investigate biomarkers such as IL-17 to determine whether specific laboratory assays can definitively assess the effects of immune checkpoint inhibitors in psoriasis patients with prior history of the disease.

## Data Availability

The original contributions presented in the study are included in the article/supplementary material. Further inquiries can be directed to the corresponding author.
